# Comparative evaluation of affibody- and antibody fragments-based CAIX imaging probes in mice bearing renal cell carcinoma xenografts

**DOI:** 10.1038/s41598-019-51445-w

**Published:** 2019-10-17

**Authors:** Javad Garousi, Fokko J. Huizing, Anzhelika Vorobyeva, Bogdan Mitran, Ken G. Andersson, Charles Dahlsson Leitao, Fredrik Y. Frejd, John Löfblom, Johan Bussink, Anna Orlova, Sandra Heskamp, Vladimir Tolmachev

**Affiliations:** 1 Department of Immunology, Genetics and Pathology, Uppsala University, Uppsala, Sweden; 2 Department of Radiation Oncology, Radboud University Medical Center, Nijmegen, The Netherlands; 3 Department of Medicinal Chemistry, Uppsala University, Uppsala, Sweden; 4 Department of Protein Science, School of Engineering Sciences in Chemistry, Biotechnology and Health, KTH Royal Institute of Technology, Stockholm, Sweden; 5 Department of Radiology and Nuclear medicine, Radboud University Medical Center, Nijmegen, The Netherlands

**Keywords:** Antibody fragment therapy, Predictive markers, Cancer imaging

## Abstract

Carbonic anhydrase IX (CAIX) is a cancer-associated molecular target for several classes of therapeutics. CAIX is overexpressed in a large fraction of renal cell carcinomas (RCC). Radionuclide molecular imaging of CAIX-expression might offer a non-invasive methodology for stratification of patients with disseminated RCC for CAIX-targeting therapeutics. Radiolabeled monoclonal antibodies and their fragments are actively investigated for imaging of CAIX expression. Promising alternatives are small non-immunoglobulin scaffold proteins, such as affibody molecules. A CAIX-targeting affibody ZCAIX:2 was re-designed with the aim to decrease off-target interactions and increase imaging contrast. The new tracer, DOTA-HE_3_-ZCAIX:2, was labeled with ^111^In and characterized *in vitro*. Tumor-targeting properties of [^111^In]In-DOTA-HE_3_-ZCAIX:2 were compared head-to-head with properties of the parental variant, [^99m^Tc]Tc(CO)_3_-HE_3_-ZCAIX:2, and the most promising antibody fragment-based tracer, [^111^In]In-DTPA-G250(Fab’)_2_, in the same batch of nude mice bearing CAIX-expressing RCC xenografts. Compared to the ^99m^Tc-labeled parental variant, [^111^In]In-DOTA-HE_3_-ZCAIX:2 provides significantly higher tumor-to-lung, tumor-to-bone and tumor-to-liver ratios, which is essential for imaging of CAIX expression in the major metastatic sites of RCC. [^111^In]In-DOTA-HE_3_-ZCAIX:2 offers significantly higher tumor-to-organ ratios compared with [^111^In]In-G250(Fab’)_2_. In conclusion, [^111^In]In-DOTA-HE_3_-ZCAIX:2 can be considered as a highly promising tracer for imaging of CAIX expression in RCC metastases based on our results and literature data.

## Introduction

Targeting of gene products that are aberrantly expressed (e.g. overexpressed) in cancer has refined treatment of disseminated cancers by increasing efficacy and reducing toxicity to normal tissues. One promising cancer-associated drug target is carbonic anhydrase IX (CAIX)^1–3^. CAIX is a membrane-bound cell-surface enzyme, which catalyzes conversion of carbon dioxide to bicarbonate and participates in regulation of intracellular and extracellular pH^4^. By acidity regulation, CAIX reduces adhesion of cancer cells and promotes their migration and invasion, thus enhancing their malignant behavior^3^.

CAIX is normally expressed on basolateral membranes of proliferating enterocytes in intestinal mucosa^5^. Expression of CAIX is mediated by the hypoxia inducible factor-1 α (HIF1α). In normoxic condition, the presence of HIF1α is tightly regulated by the von Hippel-Lindau protein (pVHL), which binds hydroxylated HIF1α followed by proteasome degradation of the whole complex. In hypoxic conditions, HIF1α is not hydroxylated and consequently it cannot be recognized by pVHL and starts accumulating in the cell. This results in overexpression of CAIX in hypoxic tumor areas^6^. It has to be noted that the hypoxia-mediated mechanism of CAIX expression is not the only one. The pVHL gene is inactivated in a large fraction of renal cell carcinomas (RCC)^7,8^, resulting in constitutive overexpression of CAIX in these tumors.

Currently, several approaches aimed at development of anti-cancer therapeutics targeting CAIX are under evaluation, including small molecule CAIX inhibitors^9,10^, small-molecule-drug conjugates^11,12^, anti-CAIX monoclonal antibodies^13,14^, anti-CAIX antibody-drug conjugates^15^, and anti-CAIX monoclonal antibodies labeled with cytotoxic radionuclides for targeted radionuclide therapy^16,17^. A sufficiently high expression of CAIX is a precondition for successful application of such therapeutics. However, CAIX has a heterogeneous expression pattern. The overexpression frequency is high in clear cell RCCs (99%), but the fraction of CAIX-expressing tumors is lower in granular cell and mixed cell RCCs (65–75%)^18^. Therefore, a reliable method for detection of CAIX in metastases is needed.

Radionuclide molecular imaging of CAIX-expression might offer a non-invasive methodology for stratification of patients with disseminated RCC for CAIX-targeting therapeutics. Furthermore, it might be useful for other applications, such as vascular endothelial growth factor (VEGF)-targeted therapy, where response to sorafenib therapy correlates with CAIX-expression level^19^. Another important area of application is based on the strong correlation between CAIX-expression and hypoxia^20–22^. High expression of CAIX might be a marker for radioresistant hypoxic cells, suggesting an appropriate adjustment of external beam radiation therapy or adding a radiosensitizing drug^23,24^.

The potential of an ^131^I-labeled anti-CAIX monoclonal antibody, G250, was evaluated for imaging of renal cell carcinoma as early as in 1993^25^. [^131^I]I-mAb G250 demonstrated considerable potential as an imaging agent in RCC patients. However, the murine origin of mAb G250 resulted in appreciable formation of human anti-mouse antibodies, which restricted its use to a single infusion^26^. A chimeric variant, cG250, was developed to solve the immunogenicity issue^13,27,28^. However, the main issue with intact monoclonal antibodies in molecular imaging is their long residence in blood, which is associated with a high background and low contrast of imaging. In addition, due to the long residence time, imaging must be performed several days after injection. Residence time of Fab and (Fab’)_2_ fragments is appreciably shorter than the residence time of intact IgGs^29^ and the affinity of (Fab’)_2_ fragments is typically higher than for Fab fragments due to avidity effect^30–32^. Radiolabeled (Fab’)_2_ fragments of cG250 (G250(Fab’)_2_) have been evaluated in tumor-bearing mice and shown to be a promising tracer for imaging of hypoxia-related CAIX expression in head-and-neck tumors^30–32^. This probe provided an optimal imaging already at 24 h after injection.

We have previously developed an affibody-based probe for *in vivo* imaging of CAIX expression. Affibody molecules are engineered affinity proteins, based on a stable three helical bundle structure^33^. The robust scaffold enables selection of binders to desirable molecular targets with high affinities (typically in the range from pM to low nM). The small size (7–8 kDa) of affibody molecules results in a rapid localization in tumors^33^. A number of affibody molecules with high affinity to cancer-associated targets have been developed and demonstrates very promising features as probes for radionuclide molecular imaging, both in preclinical and clinical studies^34^. The feasibility of affibody-mediated imaging of CAIX expression was demonstrated using a ^99m^Tc-labeled affibody molecule, ZCAIX:1^35^. Imaging properties of four different anti-CAIX affibody molecules, which were labeled with [^99m^Tc]Tc(CO)_3_ and with ^125^I via direct iodination, were compared in a follow-up study^36^. It was found that [^99m^Tc]Tc(CO)_3_-HE_3_-ZCAIX:2 should provide the best imaging of CAIX-expression in disseminated cancer^36^. However, the labeling with [^99m^Tc]Tc(CO)_3_ required a laborious multistep procedure, which might be an obstacle for clinical translation. It would be desirable to replace it with more straightforward labeling procedures, permitting potentially a kit formulation. Based on our experience with development of affibody molecules for imaging of HER2^37–39^, we selected an approach based on site-specific conjugation of DOTA (1,4,7,10-tetraazacyclododecane-1,4,7,10-tetraacetic acid) chelator at C-terminus. Introduction of a single C-terminal cysteine in ZCAIX:2 creates a unique thiol group, enabling thiol-directed coupling of maleimide-derivative of DOTA. This versatile chelator permits stable labeling with a variety of nuclides, including ^111^In for SPECT or ^68^Ga for PET^40^. We decided to keep the histidine-glutamate-histidine-glutamate-histidine-glutamate (HE_3_ or HEHEHE) tag on the N-terminus of ZCAIX:2 because addition of this tag improves biodistribution of affibody molecules^41,42^.

The goal of this study was to perform a direct comparison of imaging properties of the newly designed radiolabeled DOTA-ZCAIX:2 with the currently best available imaging probes, [^99m^Tc]Tc(CO)_3_-HE_3_-ZCAIX:2 and [^111^In]In-DTPA-G250(Fab’)_2_, to select the best variant for detection of CAIX expression in disseminated renal cell carcinoma. For this purpose, ZCAIX:2 containing a unique C-terminal cysteine was produced and site-specifically conjugated with the maleimide derivative of DOTA. DOTA-ZCAIX:2 was labeled with ^111^In and characterized *in vitro*. The biodistribution of [^111^In]In-DOTA-ZCAIX:2, [^99m^Tc]Tc(CO)_3_-HE_3_-ZCAIX:2 and [^111^In]In-DTPA-G250(Fab’)_2_ was measured in the same batch of immunodeficient mice bearing SK-RC-52 renal cell carcinoma xenografts.

## Results

### Production of DOTA-HE_3_-ZCAIX:2

**Figure 1:**
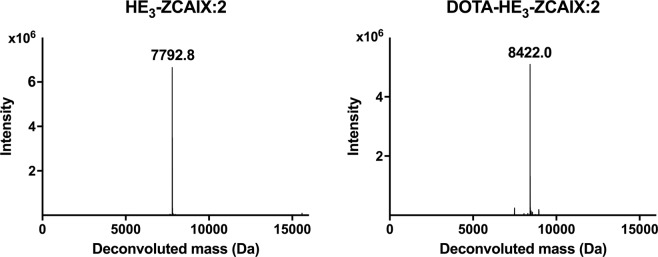
Mass-spectra deconvolution for HE_3_-ZCAIX:2 (left) and DOTA-HE_3_-ZCAIX:2 (right). The observed molecular weights of 7792 and 8422 Da, respectively, were in excellent agreement with the theoretical values (7793.5 and 8423.21 Da, respectively, calculated using https://web.expasy.org/protparam/tool).

The CAIX-binding affibody molecule HE_3_-ZCAIX:2-C containing a HEHEHE-tag at N-terminus and a cysteine at C-terminus was recombinantly produced in *Escherichia coli* The protein was conjugated to maleimide derivatives of DOTA, and the conjugate was purified to homogeneity by RP-HPLC. The molecular weight of the proteins used for labeling was confirmed using mass spectrometry (Fig. 1). The purity of DOTA-HE_3_-ZCAIX:2 exceeded 98%, as determined by analytical RP-HPLC. Molecular mass determination with electrospray ionization mass spectrometry (ESI-MS) confirmed the identity of DOTA-HE_3_-ZCAIX:2 (Fig. 1).

**Figure 2:**
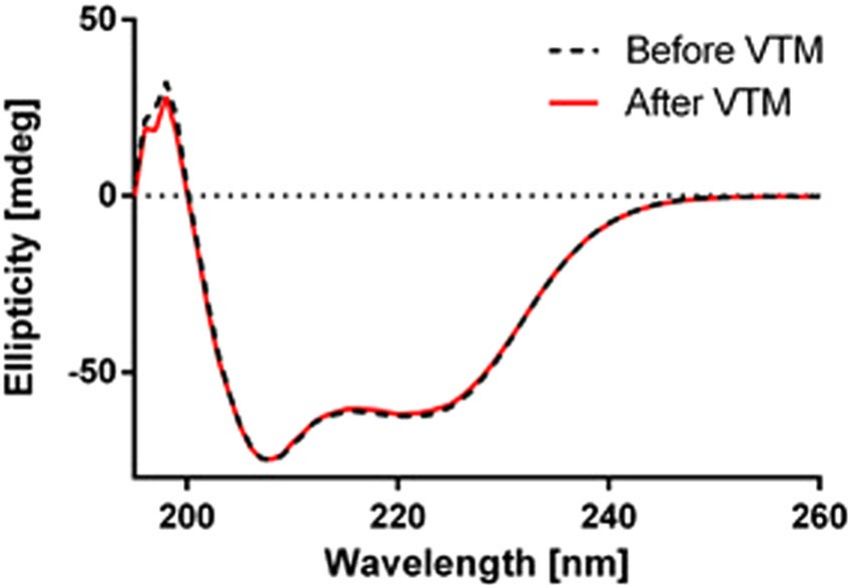
CD measurements of secondary structure of DOTA-HE_3_-ZCAIX:2 before and after warming to 90 °C.

Circular dichroism spectroscopy (Fig. 2) confirmed an alpha-helical content that is typical for affibody molecules and complete refolding of DOTA-HE_3_-ZCAIX:2 after heat-induced denaturation at 90 °C.

### Radiolabeling

DOTA-HE_3_-ZCAIX:2 was labeled with ^111^In with a radiochemical yield of 96.1 ± 2.3%. The purity of the conjugate after NAP-5 purification was 99.7 ± 0.4%. The identity of [^111^In]In-DOTA-HE_3_-ZCAIX:2 was confirmed using radio-HPLC. No release of ^111^In was observed after incubation of [^111^In]In-DOTA-HE_3_-ZCAIX:2 with 5000-fold excess of Na_4_EDTA for 2 hours at room temperature.

The isolated yield of [^99m^Tc]Tc(CO)_3_-HE_3_-ZCAIX:2 was 77 ± 2.8% and radiochemical purity was 99.7%. The isolated yield of [^111^In]In-G250(Fab’)_2_ was 73 ± 14% and radiochemical purity was 98.3 ± 0.4%.

### *In vitro* characterization of [^111^In]In-DOTA-HE_3_-ZCAIX:2

**Figure 3:**
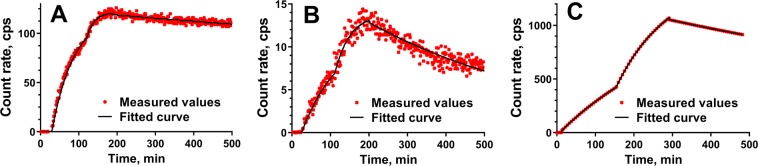
Representative LigandTracer sensorgrams of [^111^In]In-G250(Fab’)_2_ (**A**), [^111^In]In-DOTA-HE_3_-ZCAIX:2 (**B**) and [^99m^Tc]Tc(CO)_3_-HE_3_-ZCAIX:2 (**C**) binding to CAIX-expressing SK-RC-52 cells.

Affinity of [^111^In]In-DOTA-HE_3_-ZCAIX:2, [^111^In]In-G250(Fab’)_2_, and [^99m^Tc]Tc(CO)_3_-HE_3_-ZCAIX:2 binding to CAIX-expressing living SK-RC-52 cells was measured using LigandTracer. Representative LigandTracer sensorgrams are presented in Fig. 3. Both [^111^In]In-DOTA-HE_3_-ZCAIX:2 and [^111^In]In-G250(Fab’)_2_ showed rapider binding to the cells compared to [^99m^Tc]Tc(CO)_3_-HE_3_-ZCAIX:2, and the dissociation rate of [^99m^Tc]Tc(CO)_3_-HE_3_-ZCAIX:2 was slightly slower than the rate of [^111^In]In-DOTA-HE_3_-ZCAIX:2. The dissociation of [^111^In]In-G250(Fab’)_2_ was noticeable slower compared with the dissociation of both affibody molecules. The apparent equilibrium dissociation constants were calculated to be 0.12 ± 0.05 nM, 1.2 ± 0.5 nM and 6.13 ± 0.03 nM for [^111^In]In-G250(Fab’)_2_, [^111^In]In-DOTA-HE_3_-ZCAIX:2 and [^99m^Tc]Tc(CO)_3_-HE_3_-ZCAIX:2 respectively.

**Figure 4:**
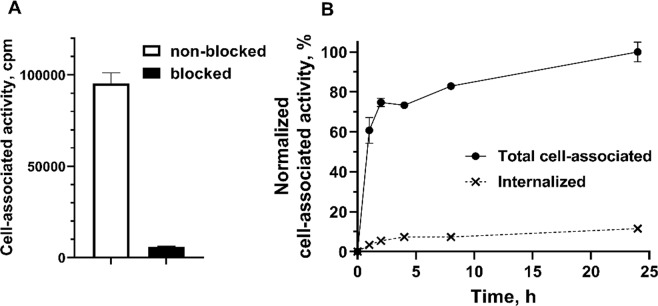
(**A**) *In vitro* specificity of [^111^In]In-DOTA-HE_3_-ZCAIX:2 binding to renal cell carcinoma SK-RC-52 cell line. In blocking group, receptors were pre-saturated by 100-fold excess of nonlabeled HE_3_-ZCAIX:2. (**B**) Internalization of [^111^In]In-DOTA-HE_3_-ZCAIX:2 by renal cell carcinoma SK-RC-52 cells during continuous incubation. Cells were incubated with conjugate (10 nM) at 37 °C. Data are normalized to the highest cell-bound activity and presented as mean values from 3 cell dishes and SD. Error bars might be not seen because they are smaller than symbols.

The results of [^111^In]In-DOTA-HE_3_-ZCAIX:2 binding specificity *in vitro* test are presented in Fig. 4A. Adding a large excess of non-labeled affibody molecule resulted in a highly significant (p < 5 × 10^−7^) reduction of cell-associated activity. This demonstrates a saturable character of binding and indicates specificity for CAIX.

Data concerning processing of [^111^In]In-DOTA-HE_3_-ZCAIX:2 by SK-RC-52 cells during continuous incubation are presented in Fig. 4B. The binding and processing pattern for [^111^In]In-DOTA-HE_3_-ZCAIX:2 was similar to the pattern for [^99m^Tc]Tc(CO)_3_-HE_3_-ZCAIX:2^36^, i.e. rapid initial binding followed by a more slow increase of cell-associated activity, and rather slow internalization. The internalized fraction at 24 h for [^111^In]In-DOTA-HE_3_-ZCAIX:2 (12 ± 1%) was smaller than for [^99m^Tc]Tc(CO)_3_-HE_3_-ZCAIX:2 (25 ± 4%), which suggests slower internalization.

### Animal studies

**Figure 5:**
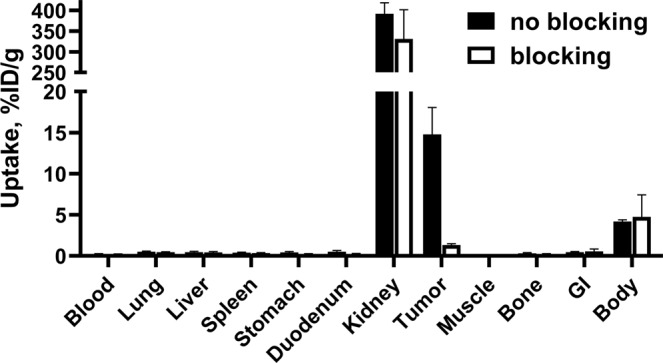
Specificity of targeting of CAIX-expressing SK-RC-52 xenografts in mice using [^111^In]In-DOTA-HE_3_-ZCAIX:2. Blocked group was subcutaneously preinjected with a 100-fold excess amount of unlabeled HE_3_-ZCAIX:2. Results are presented as the mean values for 4 mice and standard deviation.

Accumulation of [^111^In]In-DOTA-HE_3_-ZCAIX:2 in CAIX-expressing xenografts in mice was highly specific (Fig. 5). Pre-saturation of CAIX with a large amount of unlabeled HE_3_-ZCAIX:2 resulted in significantly (p < 0.0005) lower tumor uptake of the tracer (1.3 ± 0.2% ID/g) compared with the uptake without pre-saturation (15 ± 3% ID/g).

Data concerning biodistribution of [^111^In]In-DOTA-HE_3_-ZCAIX:2, [^99m^Tc]Tc(CO)_3_-HE_3_-ZCAIX:2 and [^111^In]In-G250(Fab’)_2_ are presented in Table 1.

**Table 1:** Biodistribution of radiolabeled imaging probes in BALB/C nu/nu mice bearing SK-RC-52 OV xenografts.

	[^111^In]In-DOTA-HE_3_-ZCAIX:2	[^99m^Tc]Tc(CO)_3_-HE_3_-ZCAIX:2	[^111^In]In-G250(Fab’)_2_	[^111^In]In-G250(Fab’)_2_
	4 h	4 h	4 h	24 h
Blood	0.24 ± 0.03^a^	0.31 ± 0.03^d,e^	2.9 ± 0.5^b^	0.07 ± 0.02^c^
Lung	0.49 ± 0.09	0.44 ± 0.03^d,e^	2.3 ± 0.2^b^	0.7 ± 0.3
Liver	0.5 ± 0.1^a^	0.9 ± 0.2^d,e^	10 ± 2^b^	8 ± 2^c^
Spleen	0.40 ± 0.06	0.30 ± 0.04^d,e^	10 ± 3^b^	8 ± 1^c^
Stomach	0.4 ± 0.1	0.33 ± 0.06^d^	1.3 ± 0.3^b^	0.9 ± 0.5
Duodenum	0.5 ± 0.2	0.39 ± 0.07^d,e^	3 ± 1^b^	1.5 ± 0.6^c^
Kidney	392 ± 26^a^	178 ± 17^e^	216 ± 30^b^	145 ± 9^c^
Tumor	15 ± 3^a^	7 ± 1^e^	6 ± 1^b^	5 ± 1^c^
Muscle	0.14 ± 0.02	0.12 ± 0.01^d,e^	0.8 ± 0.1^b^	0.4 ± 0.1^c^
Bone	0.32 ± 0.08	0.25 ± 0.03^d,e^	2.1 ± 0.4^b^	1.4 ± 0.2^c^
GI tract*	0.45 ± 0.07^a^	5.2 ± 0.8^d,e^	2.5 ± 0.2^b^	1.4 ± 0.3^c^

Results are presented as %ID/g (the mean values and standard deviation for four mice). Data for intestines with content are presented as % ID/whole sample.

^a^Sizgnificant difference (p < 0.05) between [^111^In]In-DOTA-ZCAIX:2 and [^99m^Tc]Tc(CO)_3_-HE_3_-ZCAIX:2;

^b^Significant difference (p < 0.05) between [^111^In]In-DOTA-ZCAIX:2 and [^111^In]In-G250(Fab’)_2_ (4 h);

^c^Significant difference (p < 0.05) between [^111^In]In-DOTA-ZCAIX:2 and [^111^In]In-G250(Fab’)_2_ (24 h);

^d^Significant difference (p < 0.05) between [^99m^Tc]Tc(CO)_3_-HE_3_-ZCAIX:2 and [^111^In]In-G250(Fab’)_2_ (4 h);

^e^Significant difference (p < 0.05) between [^99m^Tc]Tc(CO)_3_-HE_3_-ZCAIX:2 and [^111^In]In-G250(Fab’)_2_ (24 h).

The uptake of [^111^In]In-DOTA-HE_3_-ZCAIX:2 in tumors at 4 h after injection was higher than the uptake of both [^99m^Tc]Tc(CO)_3_-HE_3_-ZCAIX:2 and [^111^In]In-G250(Fab’)_2_. All imaging probes had high uptake in kidneys. Clearance of [^111^In]In-DOTA-HE_3_-ZCAIX:2 from blood was significantly quicker compared with clearance of [^99m^Tc]Tc(CO)_3_-HE_3_-ZCAIX:2. Additionally, the ^111^In-labeled affibody molecule had significantly lower uptake in liver. Uptake of [^99m^Tc]Tc(CO)_3_-HE_3_-ZCAIX:2 was lower in kidneys and in muscle. At 4 h after injection, the uptake of [^111^In]In-G250(Fab’)_2_ in majority of organs and tissues was significantly higher than the uptake of radiolabeled affibody molecules. By 24 h after injection of [^111^In]In-G250(Fab’)_2_, the activity was reduced significantly in blood, lungs, muscles and bones. The most pronounced was the reduction of the blood-borne activity.

The biodistribution features were translated into differences in tumor-to-organ ratios **(**Table 2). [^111^In]In-DOTA-HE_3_-ZCAIX:2 provided the highest tumor-to-organ ratios already at 4 h after injection. The tumor-to-organ ratios for [^111^In]In-DTPA-G250(Fab’)_2_ were expectedly the lowest at this time point. At 24 h, the tumor-to-blood, tumor-to-lung and tumor-to-muscle ratios for [^111^In]In-DTPA-G250(Fab’)_2_ increased significantly. The tumor-to-blood ratio was similar to that of [^111^In]In-DOTA-HE_3_-ZCAIX:2 at 4 h after injection. Still, the majority of tumor-to-organ ratios were higher for the radiolabeled affibody molecules at 4 h after injection compared with [^111^In]In-DTPA-G250(Fab’)_2_.

**Table 2:** Tumor-to-organ ratios of in BALB/C nu/nu mice bearing SK-RC-52 OV xenografts.

	[^111^In]In-DOTA-HE_3_-ZCAIX:2	[^99m^Tc]Tc(CO)_3_-HE_3_-ZCAIX:2	[^111^In]In-DTPA-G250(Fab’)_2_	[^111^In]In-DTPA-G250(Fab’)_2_
	4 h	4 h	4 h	24 h
Blood	63 ± 11^a^	23 ± 2^d,e^	2.1 ± 0.2^b^	67 ± 12
Lung	30 ± 3^a^	16 ± 2^d,e^	2.7 ± 0.4^b^	7 ± 1^c^
Liver	33 ± 2^a^	8 ± 2^d,e^	0.7 ± 0.3^b^	0.6 ± 0.2^c^
Spleen	37 ± 3^a^	24 ± 6^d,e^	0.7 ± 0.2^b^	0.6 ± 0.2^c^
Stomach	36 ± 4^a^	22 ± 6^d,e^	4.4 ± 0.6^b^	6 ± 3^c^
Duodenum	31 ± 8	19 ± 6^d,e^	2.4 ± 1.2^b^	3 ± 2^c^
Kidney	0.038 ± 0.008	0.04 ± 0.01^d^	0.029 ± 0.005	0.03 ± 0.01
Muscle	102 ± 20^a^	62 ± 6^d,e^	7.9 ± 0.7^b^	11 ± 1^c^
Bone	47 ± 8^a^	28 ± 3^d,e^	3.0 ± 0.5^b^	4 ± 1^c^

Results are presented as %ID/g (the mean values and standard deviation for four mice).

^a^Significant difference (p < 0.05) between [^111^In]In-DOTA-ZCAIX:2 and [^99m^Tc]Tc(CO)_3_-HE_3_-ZCAIX:2;

^b^Significant difference (p < 0.05) between [^111^In]In-DOTA-ZCAIX:2 and [^111^In]In-G250(Fab’)_2_ (4 h);

^c^Significant difference (p < 0.05) between [^111^In]In-DOTA-ZCAIX:2 and [^111^In]In-G250(Fab’)_2_ (24 h);

^d^Significant difference (p < 0.05) between [^99m^Tc]Tc(CO)_3_-HE_3_-ZCAIX:2 and [^111^In]In-G250(Fab’)_2_ (4 h);

^e^Significant difference (p < 0.05) between [^99m^Tc]Tc(CO)_3_-HE_3_-ZCAIX:2 and [^111^In]In-G250(Fab’)_2_ (4 h).

**Figure 6:**
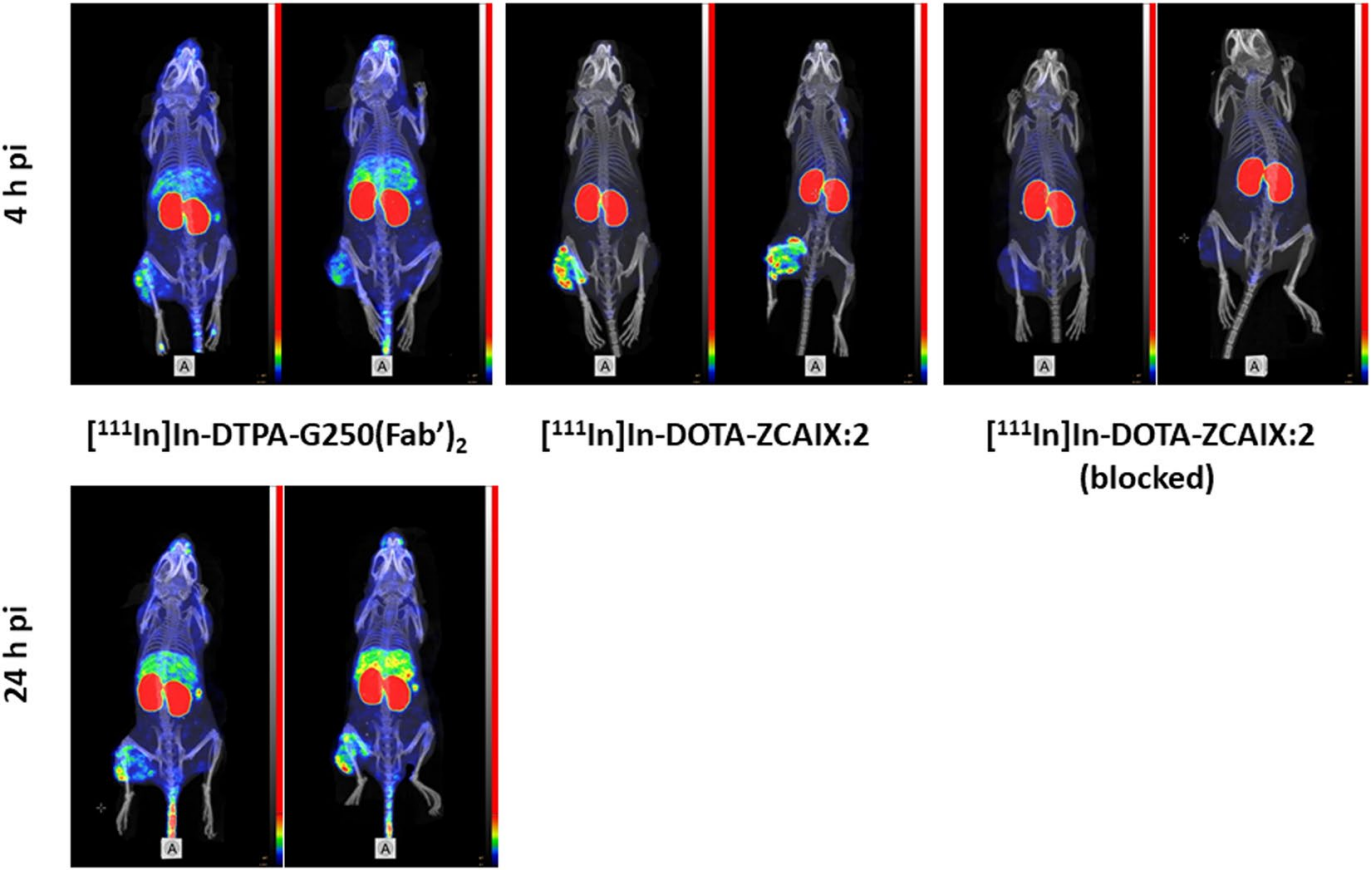
SPECT/CT imaging of CAIX-expressing SK-RC-52 xenografts using [^111^In]In-DOTA-ZCAIX:2 and [^111^In]In-DTPA-G250(Fab’)_2_. Images are presented as maximum intensity projection (MIP) in RGB (red, green and blue) color scale.

SPECT/CT imaging of CAIX-expressing SK-RC-52 xenografts using [^111^In]In-DOTA-HE_3_-ZCAIX:2 and [^111^In]In-DTPA-G250(Fab’)_2_ (Fig. 6) confirmed the results of the biodistribution experiments. SPECT showed high tumor-normal tissue contrast for [^111^In]In-DOTA-HE_3_-ZCAIX:2 at 4 h after injection. Saturation of CAIX with unlabeled affibody molecule resulted in a noticeably lower tumor uptake. [^111^In]In-DTPA-G250(Fab’)_2_ was capable to visualize tumors at both 4 and 24 h, but the contrast was higher at the second time point. Still, the contrast provided by [^111^In]In-DOTA-HE_3_-ZCAIX:2 at 4 h was higher than the contrast provided by [^111^In]In-DTPA-G250(Fab’)_2_ at 24 h after injection.

## Discussion

Genetic interpatient and intratumour heterogeneity is a serious issue for targeted anticancer therapy^43^. In the case of disseminated disease, biopsy sampling of all metastases is often unrealistic, which prompts for alternative approaches. Radionuclide molecular imaging might offer a solution in this case. However, the sensitivity of molecular imaging diagnostics must be sufficiently high. The major issue is imaging of small metastases, with a size comparable or smaller than the spatial resolution of an imaging camera. Detection of such small metastases requires a high absolute tumour uptake and contrast with normal tissue, especially at the main metastatic sites^44^. For RCC, metastases are most frequently observed in lungs and bone, and somewhat less frequently in liver and brain^45,46^. Accordingly, a probe for visualization of CAIX expression in RCC metastases should have maximal tumor-to-lung, tumor-to-bone, and tumor-to-liver ratios. High tumor-to-blood ratio is always desirable to reduce the background that is due to the blood-borne activity. Thus, development of very good binders with high affinity and high tumor uptake is not enough; we have to minimize the uptake in critical tissues by reduction of off-target interactions. We took into account these considerations when planning further improvement of an affibody-based tracer for imaging of CAIX in disseminated RCC. Furthermore, a simple and robust labeling procedure is essential to ensure successful clinical translation.

To simplify the labeling, we intended to replace a multistep procedure involving the use of [^99m^Tc]Tc(CO)_3_^+^ by more straightforward labeling with ^111^In. Our experience with affibody molecules for imaging of HER2 expression suggests that increasing the overall hydrophilicity is generally an efficient approach to reduce off-target interactions^41,47^. The use of the aminocarboxylate chelator DOTA increases the overall hydrophilicity of the affibody molecule. Besides, DOTA is a very versatile chelator, permitting labeling with a variety of nuclides suitable for radionuclide imaging. Still, a modification of a small targeting protein is always associated with a risk of undesirable effects, such as decreased affinity or poor refolding after labeling in non-physiologic conditions.

In the case of DOTA-HE_3_-ZCAIX:2, the site-specific conjugation of maleimide-DOTA did not negatively affect refolding (Fig. 2). Labeling of DOTA-HE_3_-ZCAIX:2 with ^111^In was more straightforward and two-fold quicker compared with [^99m^Tc]Tc(CO)_3_-HE_3_-ZCAIX:2, and provided a stable coupling of the radionuclide. After labeling, [^111^In]In-DOTA-HE_3_-ZCAIX:2 retained specific binding to CAIX-expressing cells *in vitro* (Fig. 4A). Affinity of [^111^In]In-DOTA-HE_3_-ZCAIX:2 to living SK-RC-52 cells was high, 1.2 ± 0.5 nM, which is higher than for [^99m^Tc]Tc(CO)_3_-HE_3_-ZCAIX:2 (6.13 ± 0.03 nM) measured using the same technique. It has to be noted that the affinity of [^111^In]In-G250(Fab’)_2_ was even higher (0.12 ± 0.05 nM), which is most likely due to avidity effects from bivalent binding. However, affinity in the single digit nanomolar range is sufficient for successful imaging of targets with high expression, such as CAIX in RCC^36^.

To compare imaging properties of the newly designed [^111^In]In-DOTA-HE_3_-ZCAIX:2, the previous best affibody-based tracer [^99m^Tc]Tc(CO)_3_-HE_3_-ZCAIX:2 and the best antibody-based tracer [^111^In]In-G250(Fab’)_2_ were selected. Their biodistribution was measured in the same batch of mice bearing SK-RC-52 xenografts. This was done to minimize batch-to-batch variability in mice physiology and xenograft quality. Accumulation of [^111^In]In-DOTA-HE_3_-ZCAIX:2 in SK-RC-52 was highly specific (Fig. 5). During this head-to-head comparison, the tumor uptake of [^111^In]In-DOTA-HE_3_-ZCAIX:2 at 4 h after injection was two-fold higher than the tumor uptake of [^99m^Tc]Tc(CO)_3_-HE_3_-ZCAIX:2 (Table 1). Most likely, the difference in tumor uptake could be explained by higher affinity of the ^111^In-labeled variant. Internalization of both [^111^In]In-DOTA-HE_3_-ZCAIX:2 (Fig. 4B) and [^99m^Tc]Tc(CO)_3_-HE_3_-ZCAIX:2^36^ after binding to cancer cells is slow. Therefore, the high affinity permits better retention of the labeled probe on the surface of cells in the tumor while the tracer is cleared from non-specific compartments. In addition, the new tracer design provided more rapid clearance from blood and lower uptake in liver compared to [^99m^Tc]Tc(CO)_3_-HE_3_-ZCAIX:2 (Table 1). Taken together with the higher tumor uptake, the new tracer provided approximately two-fold higher tumor-to-blood, tumor-to-lung, tumor-to-bones, and tumor-to-liver ratios, which has potential for increasing the sensitivity of imaging of CAIX expression in RCC metastases.

The use of [^111^In]In-DTPA-G250(Fab’)_2_ demonstrated earlier excellent potential for imaging of hypoxia-induced CAIX-expression in head-and-neck cancer^32,48^. This construct is appreciably larger (110 kDa) than the affibody molecules (7–8 kDa), and its clearance from blood and other tissues is slower (Table 1). The accumulation in tumor is lower compared with [^111^In]In-DOTA-HE_3_-ZCAIX:2 despite higher affinity. This might be because the rates of extravasation and diffusion in the tumor interstitium are lower for larger proteins. Both affibody-based constructs showed higher tumor-to-organ ratios than [^111^In]In-DTPA-G250(Fab’)_2_ at 4 h after injection (Table 2). At 24 h after injection, [^111^In]In-DTPA-G250(Fab’)_2_ provided a tumor-to-blood ratio of 67 ± 12. This is as good as the tumor-to-blood ratio provided by [^111^In]In-DOTA-HE_3_-ZCAIX:2 and by an order of magnitude better than the radiometal-labeled full-length G250 provided at 7 days after injection^31,49^. Still, the ratios between [^111^In]In-DTPA-G250(Fab’)_2_ uptake in tumor and in lung, bone or liver were lower than ratios for [^111^In]In-DOTA-HE_3_-ZCAIX:2. Thus, [^111^In]In-DOTA-HE_3_-ZCAIX:2 would be a better tracer for imaging of CAIX expression in RCC metastases.

During the last years, several small molecule sulfonamide derivatives were labeled with ^18^F^50,51^ and ^68^Ga^52^ for *in vivo* imaging of CAIX. During preclinical evaluation, these tracers demonstrated appreciably lower tumor uptake and tumor-to-organ ratios than the tracers evaluated in this study. To be fair, we have to mention that different tumor models were used in these studies, which complicates the comparison. A tracer composed from facetazolamide, a spacer, and a peptidic chelator was recently developed^53^. Upon labeling with ^99m^Tc, this tracer demonstrated an excellent tumor uptake (22% ID/g at 3 h after injection in SK-RC-52 xenografts) and very good tumor-to-blood ratio (approximately 70 and 100 at 3 and 6 h after injection, respectively). Unfortunately, tumor-to-liver and tumor-to-lung ratios peaked only at 4.7, and 2.1, respectively, i.e. substantially lower than for [^111^In]In-DOTA-HE_3_-ZCAIX:2.

Two other bivalent small molecule imaging probes, [^111^In]XYIMSR-01^54^ and [^64^Cu]XYIMSR-06^55^, have also demonstrated excellent targeting of SK-RC-52 xenografts (tumor uptake at 4 h of 20.8 ± 6.3 and 19.3 ± 4.5%ID/g, respectively) and high tumor-to-blood ratios at the day of injection. However, tumor-to-lung ratios were below 5 for both tracers.

It has to be noted that the use of DOTA permits labeling with the generator-produced positron-emitting radionuclide ^68^Ga. The use of this radionuclide would permit the use of PET for imaging and benefit from advantages such as better spatial resolution and quantification accuracy compared to SPECT.

It has to be noted that both G250 antibody and ZCAIX:2 affibody molecule do not cross-react with murine CAIX (Car9). This means that the difference in biodistribution and targeting properties of evaluated imaging probes was caused by their size (influencing their rates of clearance from blood, extravasation, and diffusion in extracellular space of tumor), their affinity and/or avidity to the target expressed in human tumor xenografts and their off-target interactions with normal tissues. However, their interaction with CAIX expressed in normal tissues cannot be evaluated in this model. Normal human tissue expression of CAIX was reported to be restricted to the upper gastrointestinal mucosa, bile ducts and pancreas^5^. Nevertheless, four clinical imaging studies in more than 100 patients, using radiolabeled G250 monoclonal antibodies or G250(Fab’)_2_, have not demonstrated noticeable accumulation of the tracer in these tissues^16,26–28^. Furthermore, clinical radionuclide therapy with doses stabilizing previously progressing tumors or causing some tumor shrinkage, have not caused any gastrointestinal toxicity^16,17,26^. This lead us to a conclusion that the expression in normal tissue is much lower than in tumors and would not influence imaging contrast. Thus, the murine model is adequate for assessment of CAIX targeting probes in human xenografts, although it does not permit evaluation of the effect of non-cancerous CAIX expression.

Both [^111^In]In-DTPA-G250(Fab’)_2_ and [^111^In]In-DOTA-HE_3_-ZCAIX:2 have high uptake in kidneys. In the case of [^111^In]In-DOTA-HE_3_-ZCAIX:2, the uptake is unspecific since it is not blocked be unlabeled tracer. This would not prevent affibody-mediated imaging of metastases^38^, but makes radiometal-labeled affibody molecules unsuitable for therapeutic targeting. Earlier studies with HER2-targeting affibody molecules have demonstrated that the renal uptake of affibody molecules is not mediated by megalin, and it could not be blocked by co- or preinjection of lysine or Gelofusine^56^. We have shown that the use of affibody-based bioorthogonal chemistry- or peptide nucleic acid-medicated pretargeting prevents high kidney accumulation in the case of HER2-targeting affibody molecules^57,58^ and enables successful radionuclide therapy in mice^59^. Application of this approach might also enable pretargeting radionuclide therapy of CAIX-expressing tumors.

A recent clinical study demonstrated that a combination of [^89^Zr]Zr-DFO-girentuximab-immunoPET/CT and CT detected more lesions than CT alone (91% vs 56%) and more than combination of CT and [^18^F]FDG-PET/CT (84%) in the case of clear cell RCC^60^. Thus, in principle imaging with radiolabeled DOTA-HE_3_-ZCAIX:2 might be used for staging of clear cell RCC, where expression of CAIX is high. It has to be stressed that we propose to use [^111^In]In-DOTA-HE_3_-ZCAIX:2 first and foremost for detection of CAIX expression in known RCC metastases for selection of patients for CAIX-targeting treatment, but not for detection of metastatic RCC. Since a substantial fraction of granular cell and mixed cell RCCs is CAIX-negative^18^, the use of [^111^In]In-DOTA-HE_3_-ZCAIX:2 might result in false-negative findings.

## Conclusion

Modification of labeling strategy permitted appreciable improvement of tumor uptake and tumor-to-organ ratios of a tracer based on the ZCAIX:2 affibody molecule. Based on our results and literature data, [^111^In]In-DOTA-HE_3_-ZCAIX:2 can be considered a highly promising tracer for imaging of CAIX expression in RCC metastases.

## Methods

### Reagents, equipment and statistics

[^111^In]InCl_3_ was purchased from Mallinckrodt Sweden AB (Stockholm, Sweden). [^99m^Tc]TcO_4_^−^ was obtained by elution of UltraTechneKow generator (Mallinckrodt Pharmaceuticals, Dublin, Ireland) with sterile 0.9% NaCl. The CRS kits for production of technetium tricarbonyl were purchased from Center for Radiopharmaceutical sciences (Villigen, Switzerland). An automatic gamma-spectrometer with a NaI (Tl) detector (1480 WIZARDWallac Oy, Turku, Finland) was used for activity measurement activity in cell binding and biodistribution experiments. Formulation of injection solutions was performed using VDC-405 ionization chamber (Veenstra Instruments BV, The Netherlands).

For purification, a FPLC system (GE Healthcare AKTA purifier 10 system) and for polishing a 1200 series HPLC (Agilent Technologies, Santa Clara, CA) were used. ESI-MS with a 6520 Accurate-Mass Q-TOF LC/MS (Agilent Technologies) was used for confirmation of molecular masses of the affibody molecules. Circular dichroism spectroscopy was performed using a Chirascan spectropolarimeter (Applied Photophysics, United Kingdom).

Instant thin layer chromatography (ITLC) was performed using silica gel-impregnated glass microfiber sheets (ITLC-SG strips, Varian, Lake Forest, CA). Distribution of the radioactivity on the strips was measured on Cyclone Phosphor Storage Screen using OptiQuant software for data processing (both Packard Instrument Company, Meriden, CT, US).

Statistical analysis of data was performed using GraphPad Prism (version 8.00 for Windows GraphPad Software, San Diego CA) to find any significant differences (p < 0.05). An unpaired two-tailed t-test was used for analysis if it was not stated otherwise. One-way ANOVA analysis with Bonferroni’s multiple comparison test was used to evaluate differences between more than two data sets.

### Proteins production and characterization

Anti-CAIX HE_3_-ZCAIX:2 affibody molecule was produced as described earlier^36^. A C-terminal cysteine-containing variant was produced as described earlier^61^. Briefly, the protein was produced in *E*. *coli* BL21*(DE3) (Thermo Fisher Scientific) in an overnight culture at 25 °C after induced expression with 100 μM Isopropyl β-D-1-thiogalactopyranoside (IPTG) at an OD600 of 0.8. Following cell lysis with French press, the supernatant was heated to 95 °C for 10 min with subsequent incubation on ice for 20 min, followed by centrifugation to remove precipitated proteins. Supernatant, containing affibody molecule, was purified by immobilized metal affinity chromatography (IMAC), using nickel- nitriloacetic acid (Ni-NTA) column on an ÄKTA FPLC system (GE Healthcare, Uppsala, Sweden). IMAC purification was done by running 20 mM tris-hydrochloride; 500 mM sodium chloride, pH 8 (buffer A) and 300 mM imidazole (buffer B) onto the loaded column with the filtered cell lysate. The column was washed with 30 mM imidazole followed by elution using a 30–300 mM imidazole gradient. The buffer of the eluate was changed to 20 mM ammonium acetate, pH 5.5, and the proteins were freeze-dried.

The conjugation was performed using the method described earlier^56^. The protein was dissolved in 20 mM ammonium acetate, pH 5.5, and reduced with an equimolar concentration of tris(2-carboxyethyl)phosphine (TCEP) for 30 min at 37 °C. The proteins were incubated at 37 °C for 90 min with ten-fold molar excess of maleimide derivative of DOTA for site-specific conjugation to the C-terminal cysteine. Metal ion contaminations were removed from all buffers with Chelex 100 resin (Bio-Rad Laboratories). The conjugate was purified by reverse-phase high performance liquid chromatography (RP-HPLC) using a Zorbax 300SB-C18 semi-preparative column (Agilent Technologies, Santa Clara, CA). Water with 0.1% trifluoroacetic acid was used as running buffer and an acetonitrile gradient was used for elution.

Molecular masses of both HE_3_-ZCAIX:2 and DOTA-HE_3_-ZCAIX:2 were determined using LC/MS. Circular dichroism spectroscopy was performed using a spectropolarimeter with an optical path length of 1 mm, to analyse the alpha-helical content, thermal stability and refolding capacity of DOTA-HE3-ZCAIX:2 at a concentration of 0.25 mg/mL. The thermal stability was evaluated by measuring the change in ellipticity at 221 nm during heating (5 °C/min) from 20 to 90 °C. The melting temperature (T_m_) was approximated from the data acquired from variable temperature measurements (VTM) by curve fitting using a Boltzmann Sigmoidal model (GraphPad Prism, version 7). The refolding capacity was assessed by comparing spectra obtained from measurements at wavelengths in the range 195–260 nm at 20 °C, before and after thermal denaturation.

DTPA-G250(Fab’)_2_ was produced by enzymatic digestion of the monoclonal chimeric anti-CAIX antibody girentuximab (G250 Wilex AG) with pepsine and conjugated with isothiocyanatobenzyl-diethylenetriaminepentaacetic acid (ITC-DTPA, Macrocyclis,Houston, TX, USA) as described previously^32,48^.

### Radiolabeling

Labeling of DOTA-HE_3_-ZCAIX:2 with ^111^In, the conjugate 50 µg in 75 µL 0.2 M ammonium acetate, pH 5.5, was mixed with 53 MBq (50 µl) [^111^In]InCl_3_. The mixture was incubated for 60 min at 90 °C. Thereafter, a 5000-fold excess of tetrasodium salt of ethylenediaminetetracetic acid (Na_4_EDTA) was added to the mixture, and [^111^In]In-DOTA-HE_3_-ZCAIX:2 was purified using NAP-5 columns (GE Healthcare, Uppsala, Sweden) pre-equilibrated and eluted with PBS. Purity was controlled using ITLC eluted with 0.1 M citrate buffer pH 6.0. The ITLC data were cross-validated using radio-HPLC.

To test stability of the label, [^111^In]In-DOTA-HE_3_-ZCAIX:2 was incubated with 5000-fold excess of Na_4_EDTA for 2 hours at room temperature. The mixture was analyzed using ITLC as described above.

Labeling of DTPA-G250(Fab’)_2_ with ^111^In was performed as described and validated earlier^32,48^. Briefly, a stock solution of 40 μg DTPA-G250(Fab’)_2_ (60 μL) was diluted in 120 μL 0.5 M 2-(N-morpholino)ethanesulfonic acid (MES) buffer, pH 5.4, and mixed with 60 μL (20 MBq) [^111^In]InCl_3_. The mixture was incubated for 60 min at room temperature, and [^111^In]In-G250(Fab’)_2_ was purified using NAP-5 columns pre-equilibrated and eluted with PBS. Purity was controlled using ITLC eluted with 0.1 M citrate buffer pH 6.0.

Labeling of HE_3_-ZCAIX:2 for comparative biodistribution experiment with [^99m^Tc(CO)_3_]^+^ was performed as described earlier by Garousi and co-workers^36^. Briefly, a generator eluate (400–500 μL) containing ca. 3 GBq of ^99m^Tc was added to a CRS kit and the mixture was incubated at 100 °C for 30 min. After incubation, 40 μL of mixture was transferred to a vial containing 50 μg of Affibody molecule in 40 μL of PBS. The mixture was incubated for 100 min at 50 °C. Thereafter, a 5000-fold molar excess of histidine was added to the reaction mixture, and it was incubated at 50 °C for 20 min. The radiolabeled HE_3_-ZCAIX:2 were purified using NAP-5 columns pre-equilibrated and eluted with PBS. The radiochemical yield and purity of each preparation was measured using radio-ITLC eluted with PBS.

### *In vitro* characterization of [^111^In]In-DOTA-HE_3_-ZCAIX:2

The *in vitro* characterization of [^111^In]In-DOTA-HE_3_-ZCAIX:2 was performed in the same way as earlier characterization of [^99m^Tc]Tc(CO)_3_-HE_3_-ZCAIX:2^36^. The CAIX-positive human renal cell carcinoma cell line SK-RC-52 was used as a model.

Affinity of binding was determined using LigandTracer Yellow (Ridgeview Instruments, Vänge, Sweden) as described previously^36,62^ and analyzed using InteractionMap software^63^. This device records in real time kinetic binding to and dissociation of radiolabeled tracers from living cells. The TraceDrawer Software (Ridgeview Instruments, Vänge, Sweden) was used to calculate the affinity based on the association and dissociation rates determined by adding increasing concentrations of each radioconjugate to the cell cultures followed by monitoring the retention at zero concentration. For [^111^In]In-DOTA-HE_3_-ZCAIX:2 and [^99m^Tc]Tc(CO)_3_-HE_3_-ZCAIX:2, the association was measured at concentration of 2 and 6 nM. For [^111^In]In-G250(Fab’)_2_, the association was measured at 1.5 and 4.5 nM.

To evaluate specificity and cellular processing, SK-RC-52 cells (~1 × 10^6^ cells/dish) were used. For the determining of specificity, a set of six cell dishes was used. [^111^In]In-DOTA-HE_3_-ZCAIX:2 was added at a concentration of 10 nM. Fifteen min before adding of [^111^In]In-DOTA-HE_3_-ZCAIX:2, CAIX on cells in a set of three dished was pre-saturated by non-labeled HE_3_-ZCAIX:2 to a total concentration of 1 mM. All cells were incubated for 1 h at 37 °C in a humidified incubator equilibrated with 5% CO_2_. Then, the medium was collected, the cells were washed with cold serum-free medium and detached by treatment with trypsin-EDTA solution for 10 min at 37 °C. The detached cells were collected, and the radioactivity of cells and media was measured. Binding specificity of [^99m^Tc]Tc(CO)_3_-HE_3_-ZCAIX:2 and [^111^In]In-G250(Fab’)_2_ has been confirmed earlier in a similar way^32,36^.

For evaluation of cellular processing, cells were incubated in a humidified incubator (5% CO_2_, 37 °C) with [^111^In]In-DOTA-HE_3_-ZCAIX:2 (at concentration of 10 nM). A set of three dishes was taken from the incubator at 1, 2, 4, 8 and 24 h after the incubation initiation, and membrane-bound and internalized radionuclides were discriminated using a modified acid wash method, as describe earlier^64^.

### Animal studies

Animal studies were performed according to national legislation on laboratory animal protection and were approved by the Ethical Committee for Animal Research in Uppsala.

For tumor implantation, 10^7^ of CAIX-expressing SK-RC-52 cells in 100 µL of RPMI 1640 medium were subcutaneously injected in the right hind leg of 7-weeks old female BALB/c nu/nu mice (Scanbur A/S, Karlslunde, Denmark). The experiments were performed two weeks after tumor cell implantation. The average animal weight was 16.6 ± 1.3, and the average tumor weight at dissection was 0.22 ± 0.11 g.

In the biodistribution study, four mice per data point were used. Two groups of mice were intravenously injected with 30 kBq [^111^In]In-DOTA-HE_3_-ZCAIX:2 in 100 µL PBS. The injected protein dose was adjusted to 5 µg per mouse with nonlabeled conjugate. To test *in vivo* targeting specificity, animals in one group were pre-injected with 500 µg of nonlabeled ZCAIX:2 30 min before injection of [^111^In]In-DOTA-HE_3_-ZCAIX:2 to saturate tumors. One group was injected with 30 kBq (5 µg) of [^99m^Tc]Tc(CO)_3_-HE_3_-ZCAIX:2. Two groups were injected with 30 kBq (10 µg) of [^111^In]In-G250(Fab’)_2_. Biodistribution was measured at 4 h after injection of radiolabeled affibody molecules and 4 and 24 h after injection of [^111^In]In-G250(Fab’)_2_. The mice were anesthetized by an intraperitoneal injection of a lethal dose ketamine and xylazine solution and exsanguinated by heart puncture. Blood was collected with a heparinized syringe, organs were collected, weighed and activity was measured using a gamma spectrometer. The percent of injected dose per gram of sample (%ID/g) was calculated, except for gastrointestinal tract and carcass, where %ID per whole sample was calculated.

Small animal SPECT/CT (nanoScan SC equipped with 16-pinholes collimators, Mediso Medical Imaging Systems, Hungary) imaging was performed to obtain a visual confirmation of the biodistribution data. Two mice per data point were used to check a reproducibility of the data. One set of two mice was injected with 5 MBq (5 µg) of [^111^In]In-DOTA-HE_3_-ZCAIX:2. For specificity control, another set of mice was injected with 5 MBq (500 µg) of [^111^In]In-DOTA-HE_3_-ZCAIX:2. Two sets of mice were injected with 3 MBq (10 µg) of [^111^In]In-G250(Fab’)_2_. Imaging was performed at 4 h after injection [^111^In]In-DOTA-HE_3_-ZCAIX:2 and at 4 and 24 h after injection of [^111^In]In-G250(Fab’)_2_. Immediately before imaging, mice we sacrificed by inhalation of carbon dioxide. CT scans were acquired at the following parameters: 50 keV energy peak, 670 μA, 480 projections, 5.26 min acquisition time. SPECT scanning was performed during 30 min using ^111^In gamma-peaks of 245.4 keV and 171.3 keV (window width of 20%). CT raw files were reconstructed using Nucline 2.03 Software (Mediso Medical Imaging Systems, Hungary). SPECT raw data were reconstructed using Tera-Tomo™ 3D SPECT reconstruction technology.
